# Effects of *Lacticaseibacillus casei* (*Lactobacillus casei*) and *Saccharomyces cerevisiae* mixture on growth performance, hematological parameters, immunological responses, and intestinal microbiome in weaned pigs

**DOI:** 10.3389/fvets.2023.1140718

**Published:** 2023-06-12

**Authors:** Sheena Kim, Jinok Kwak, Minho Song, Jinho Cho, Eun Sol Kim, Gi Beom Keum, Hyunok Doo, Sriniwas Pandey, Jae Hyoung Cho, Sumin Ryu, San Kim, Yu-Mi Im, Hyeun Bum Kim

**Affiliations:** ^1^Department of Animal Resources Science, Dankook University, Cheonan, Republic of Korea; ^2^Division of Animal and Dairy Science, Chungnam National University, Daejeon, Republic of Korea; ^3^Division of Food and Animal Science, Chungbuk National University, Cheongju, Republic of Korea; ^4^BRD Korea Corp., Hwaseong, Republic of Korea; ^5^Department of Nursing, Dankook University, Cheonan, Republic of Korea

**Keywords:** mixed effective microorganisms, growth performance, gut microbiome, weanling pigs, hematology

## Abstract

**Introduction:**

This study was conducted to evaluate the effects of *Lacticaseibacillus casei* (*Lactobacillus casei*) and *Saccharomyces cerevisiae* mixture on growth performance, hematological parameters, immunological responses, and gut microbiome in weaned pigs.

**Methods:**

A total of 300 crossbred pigs [(Landrace × Yorkshire] × Duroc; 8.87 ± 0.34  kg of average initial body weight [BW]; 4  weeks of age) were divided into two dietary treatments (15 pigs/pen, 10 replicates/treatment) using a randomized complete block design (block = BW): control (CON) and the effective microorganism (MEM). The CON was not treated, while the MEM was treated with the mixture of *L. casei* (1 × 10^7^ CFU/mL) and *S. cerevisiae* (1 × 10^7^ CFU/mL) at 3 mL/pig/day for 4  weeks *via* the drinking water supply. Two feces and one blood sample from the randomly selected pigs in each pen were collected on D1 and D28 after weaning. Pigs were individually weighed, and pen feed intakes were recorded to evaluate pig growth performance. For the gut microbiome analysis, 16S rRNA gene hypervariable regions (V5 to V6) were sequenced using the Illumina MiSeq platform, and Quantitative Insight into Microbial Ecology (QIIME) and Microbiome Helper pipeline were used for 16S rRNA gene sequence analysis.

**Results and Discussion:**

The daily weight gain and feed efficiency of MEM were significantly higher than those of CON (*p* < 0.001). There were no significant differences in hematological parameters and immune responses between CON and MEM. However, MEM had significantly lower *Treponema* genus, whereas significantly higher *Lactobacillus* and *Roseburia* genera compared to CON. Overall, our data showed that *L. casei* and *S. cerevisiae* mixture could promote growth performance through the modulation of gut microbiota in pigs. This study will help to understand the correlation between the growth performance and the gut microbiome.

## Introduction

Weaning is a very important period in which the piglets have to deal with separation from the sow, along with the feed changes from milk to solid feed (1, 2). Weaning imposes enormous stress on the piglets and causes the marked microbiological and physiological changes in the pig (3). As a result, the poor growth performance including slow growth rate, reduced feed intake and reduced feed conversion of post-weaning piglets can be induced by the biological stresses after weaning (4). Consequently, the weaning transition generally causes huge economic losses in the pig industry (2, 5). To overcome the weaning stresses, antibiotic growth promoters (AGPs) have previously been used to improve growth rate and to prevent diseases. However, the use of AGPs have been banned in the numerous countries including the United States and the European Union because of the increased public health concerns, such as antimicrobial resistance (6). Therefore, there has been a need for alternatives to feed antibiotics to reduce mortality and improve gut health in pigs during the pivotal weaning period.

The various alternatives to AGPs have been evaluated to enhance pig growth performance and to prevent diseases. Among them, beneficial microorganisms have been widely used in swine industry worldwide because they are known to improve the gut microbiome and the livestock productivity.

The porcine gastrointestinal tract (GIT) harbors a highly diverse microbial ecosystem (7), which, once developed, remains stable over time (8). This variety of microbiota has the profound effects on the development of immunological, morphological, and physiological development of the GIT (8). The microbiota contributes to the digestion of nutrients and forms a layer on mucosal surfaces that protects from overgrowth of pathogens (9). As advanced metagenomics using next generation sequencing have revealed high correlations between the gut microbiome and the health of animals, the roles of intestinal microbiome to improve livestock productivity and to prevent diseases are being emphasized. Various studies are being conducted on effective microorganisms that can improve the gut microbiome and the livestock productivity. Beneficial microorganisms are also commonly known to improve intestinal health as well (10). One of the commonly used beneficial bacteria in the swine industry is *Lacticaseibacillus casei* (*L. casei*) that was formerly known as *Lactobacillus casei*. *L. casei* is a dominant beneficial bacterium in the pig intestine, and is known as a strain that has been recognized for its immunomodulatory and harmful bacteria suppression functions (11). Another commonly used beneficial bacteria in the swine industry is *Saccharomyces cerevisiae* (*S. cerevisiae*) that is a yeast. *S. cerevisiae* is not a naturally occurring strain in the pig intestinal tract, but it has effects such as adsorption of toxins from β-glucan present in the cell wall, stimulation of the immune system, and suppression of pathogen attachment in the intestinal tract (12). Probiotics that contain multiple strains of different species are generally more effective than those with multiple strains of the same species because the mixed strains of different probiotic species can work together in the host gut environmental conditions (13). As an example, Giang et al. reported that a combination of yeast with lactic acid bacteria (LAB) had better probiotic effects on growth performance and digestion over a longer time period than the single use of LAB alone (14). Therefore, the combined use of these two strains (*L. casei* and *S. cerevisiae*) is expected to have a synergistic effect on the development of the intestinal tract and the establishment of the immune system during weaning. With these backgrounds, this study was conducted to determine the effects of a mixture of effective microorganisms (*L. casei* and *S. cerevisiae*) on growth performance, hematological parameters, immunological responses, and intestinal microbiome in weaned pigs.

## Materials and methods

### Animal ethics statement

The protocol used in this experiment was reviewed and approved by the Institutional Animal Care and Use Committee of Dankook University, Cheonan, South Korea (approval no. DKU-21-040). The experiment was conducted at the Saeiri-Farm, Jincheon, South Korea (Coordinates: 36° 51′ 24.00″ N / 127° 26′ 36.00″ E) in where the average temperature and precipitation during the experimental period were 20.2°C and 6.4 mm, respectively.

### Experimental design, animals, and housing

A total of 300 crossbred piglets [(Landrace × Yorkshire) × Duroc; 8.87 ± 0.34 kg of average initial body weight [BW]; 4 weeks of age] were used in this experiment. These pigs were randomly allotted to two treatments (15 pigs/pen, 10 replicates/treatment) using a randomized complete block design (block = BW): control (CON) and the effective microorganism (MEM). The animals used in the experiment were female and castrated male pigs, and both genders were randomly assigned into the respective dietary treatments. The liquid form of MEM mixture containing 1×10^7^ CFU/mL of each *L. casei* and *S. cerevisiae* was provided by the government institution, Korean Agricultural Technology Center (Jincheon, South Korea). The optimal dose of MEM was determined by Korean Agricultural Technology Center (Jincheon, South Korea), and pigs in the MEM treatment received the optimal dose of *L. casei* (1 × 10^7^ CFU/mL) and *S. cerevisiae* (1 × 10^7^ CFU/mL) at 3 mL/pig/day for 4 weeks *via* the drinking water supply using a proportional dosing pump for treatments (Dosatron^®^, Tresses, France) according to the manufacturer’s instructions. All pigs were fed a corn soybean meal based commercial basal diet formulated to meet the nutrient requirements proposed by the National Research Council (Table 1) (15). Pigs were housed in the room with slatted plastic floors, automated ventilation and heating systems. Pigs had free access to feed and water for the entire duration of the experiment.

**Table 1:** Composition of the basal diets for weaning pigs (as-fed basis).

Item^a^	CON
Ingredient, %	
Corn	46.91
Whey powder	15.00
Soybean meal, 44%	20.00
Soy protein concentrate	11.50
Soybean oil	2.90
Limestone	1.34
Monocalcium phosphate	0.95
Vitamin premix^a^	0.40
Mineral premix^b^	0.40
L-Lys∙HCl	0.34
DL-Met	0.18
L-Thr	0.08
Total	100.00
Calculated energy and nutrient	
Metabolizable energy, kcal/kg	3,465
Crude protein, %	22.36
Ca, %	0.85
P, %	0.65
Lys, %	1.54
Met, %	0.47

a Vitamin premix provided the following quantities of vitamin per kilogram of complete diet: vitamin A, 12,000 IU; vitamin D_3_, 2,500 IU; vitamin E, 40 IU; vitamin K_3_, 3 mg; D-pantothenic acid, 15 mg; nicotinic acid, 40 mg; choline, 400 mg; and vitamin B_12_, 12 μg.

b Mineral premix provided the following quantities of mineral per kilogram of complete diet: Fe, 90 mg from iron sulfate; Cu, 8.8 mg from copper sulfate; Zn, 100 mg from zinc oxide; Mn, 54 mg from manganese oxide; I, 0.35 mg from potassium iodide; Se, 0.30 mg from sodium selenite.

### Sample and data collection

For the evaluation of pig growth performance, pigs were individually weighed on day 1 (D1) and 28 (D28), and the average daily weight gain (ADG) was calculated. Feed consumption was also recorded at the same time (D1 and D28), and the average daily feed intake (ADFI) and feed efficiency (gain:feed) were calculated. Two fecal samples from the randomly selected pigs in each pen (a total of 20 samples/treatment/time point) were collected directly from the rectum on D1 and D28 after weaning. Diarrhea scores in pigs were assessed visually on a pen basis by 2 evaluators for 2 weeks after the start of the experiment as follows: 0, very hard, often pellet-like faeces; 1, well-formed faeces firm to cut; 2, formed faeces but soft to cut; 3, faeces falling out of shape upon contact with surfaces, sloppy; 4, pasty diarrhea; 5, liquid diarrhea (16). The blood samples (a total of 10 samples/treatment/time point) were collected from the jugular veins of the randomly selected pigs using vacutainer tubes of 5 mL containing with or without ethylenediaminetetraacetic acid (EDTA) as an anticoagulant on D1 and D28 of the experiment (17). The blood samples collected with EDTA were stored at 4°C until usage, then they were used for the complete blood count analysis. The blood samples collected without EDTA were left to clot at room temperature for 2 h. Then the serum samples were prepared by centrifuging the blood samples at 3,000 × g at 4°C for 15 min, and stored at −80°C until they were used for Enzyme-Linked Immunosorbent Assay (ELISA).

### Blood sample analysis

Hematological parameters were analyzed using the Scil Vet abc hematology analyzer (Scil Animal Care Company, Altorf, France) that was calibrated for porcine blood. The concentrations of serum cortisol, tumor necrosis factor-α (TNF-α) (R&D Systems, Inc., Minneapolis, United States), and immunoglobulin G, M, and A (Abnova Corp., Taipei City, Taiwan) were determined using the ELISA kits according to the manufacturer’s protocol.

### 16S rRNA gene analysis

For the total DNA extraction from the feces, 200 mg of feces per sample was used and the DNA extraction was conducted using the QIAamp Fast DNA Stool Mini Kit (QIAGEN, Hilden, Germany) following the manufacturer’s protocol. The Colibri Microvolume Spectrometer (Titertek Berthold, Pforzheim, Germany) was used to measure the concentrations of DNA, and the samples with OD260/280 ratios of 1.80–2.15 were utilized further. To amplify the V5 to V6 hypervariable regions of the 16S rRNA genes, the PCR primer sets consisted of 799F-mod6 (5’ CMGGATTAGATACCCKGGT-3′) and 1114R (5’-GGGTTGCGCTCGTTGC-3′) were used. Briefly, 25 ng of DNA in a reaction volume of 50 μL was used, and the amplification mix contained 5X PrimeSTAR Buffer (Mg2+) (Takara Bio, Inc., Shiga, Japan), 2.5 mM concentrations of each deoxynucleotide triphosphates, 2.5 U/μL of PrimeSTAR HS DNA Polymerase, and 10 pmol of each primer. The polymerase chain reaction cycling conditions were as follows: initial denaturation was at 98°C for 3 min, followed by 30 cycles of 98°C for 10 s, 55°C for 15 s, and 72°C for 30 s, and a final 3-min extension at 72°C. Then, the PCR product was purified using Wizard^®^ SV Gel and PCR Clean Up System purification kit (Promega, Madison, United States) according to the manufacturer’s instructions. The sequencing of the 16S rRNA gene amplicons was conducted using the Illumina MiSeq chemistry at BRD Inc. (Dongtan, Republic of Korea) according to the manufacturer’s instructions. All the raw sequence data generated from the Illumina MiSeq platform were quality checked utilizing FastQC (18, 19). Then, 16S rRNA gene sequences were analyzed using the Deblur algorithm implemented in the QIIME2 and Microbiome Helper pipeline.

### Statistical analysis

Data were analyzed using the General Linear Model Procedure of SAS (version 9.0, SAS Inst. Inc., Cary, United States) in a randomized complete block design with the initial BW as a block, considering the pen as the experimental unit. The statistical model for growth performance, hematological parameters and immunological responses included the effect of dietary treatments as a fixed effect. The statistical significance was set as the *p* value of less than 0.05 (*p* < 0.05). The results were presented as the mean ± standard error of the mean (SEM). Alpha diversity indices, such as Shannon, Simpson, observed OTUs, and Chao1 of each dietary treatment were calculated utilizing the MicrobiomeAnalystR. Significant differences in alpha diversity indices between dietary treatments were calculated based on ANOVA. To determine significant differences in beta diversity, statistical comparisons of weighted and unweighted UniFrac distances between dietary treatments were performed using analysis of similarities (ANOSIM).

## Results and discussion

### Growth performance

There were no differences on final BW and ADFI of pigs during the experimental period between dietary treatments (Table 2). However, the daily weight gain (279 g/d vs. 222 g/d) and feed efficiency (0.62 g/g vs. 0.52 g/g) of weanling pigs were significantly higher in MEM than those in CON (*p* < 0.001). During the first 14 days after weaning, no treatment effects were observed in the diarrhea scores between CON and MEM (Table 2). The growth performance observed in this study agrees with previous results, and showed that the overall growth performance was improved when useful microorganisms were fed. In the previous study, the *L. casei* fed group showed a higher weight gain than the control group, and a pattern similar to that of the antibiotic (colistin sulfate) group was observed for growth performance (20). Jang et al. reported that there was no significant difference in diarrhea frequency compared to the control group when probiotics (*L. casei*, *Bacillus subtilis*, *Lactobacillus crispatus*) were fed, but the daily weight gain showed a tendency to improve by 4% and feed efficiency by 6% (21). However, Francisco Tortuero reported that there was no difference in the growth of weaned pigs compared to the control group when mixed with *Streptococcus faecium* M74 (22). Because of these discrepancies between results from previous studies, it was suggested that the growth promoting and diarrhea preventing effects of probiotic bacteria should be observed when pigs were exposed to stress or had health problems, resulting in a greater effects (23, 24). Kritas et al., reported that it was effective in improving prevalence and growth performance in pigs infected with porcine reproductive and respiratory syndrome virus, although it did not show weight improvement under normal conditions (24). Similarly, in a previous study*, L. casei* treatment was reported to improve the diarrhea index in pigs infected with pathogenic bacteria (23). Wang et al., also reported that *L. casei* plays an important role in contributing to intestinal morphological development by preventing intestinal pathological damage and reducing inflammation (11).

**Table 2:** Effect of mixture of effective microorganisms on growth performance parameters^a^ and diarrhea score of piglets.

Item^b^	CON	MEM	SEM	*p*-value
Initial BW, kg	9.11	8.53	0.88	0.102
Final BW, kg	15.55	16.61	0.40	0.087
ADG, g/d	222	279	4.97	< 0.001
ADFI, g/d	428	447	7.17	0.094
Feed efficiency, g/g	0.52	0.62	0.01	< 0.001
Diarrhea score^c^	3.49	3.45	0.34	0.746

a Pigs were individually weighed, and pen feed intakes were recorded to evaluate pig growth performance on D1 and D28. Each value is the mean of 10 replicates.

b CON, a control diet without any treatments; MEM, a diet treated with mixture of effective microorganisms; SEM, standard error of mean.

c Diarrhea scores in pigs were assessed visually on a pen basis by 2 evaluators for 2 weeks after the start of the experiment. Diarrhea score: 0 = very hard, often pellet-like faeces; 1 = well-formed faeces firm to cut; 2 = formed faeces but soft to cut; 3 = faeces falling out of shape upon contact with surfaces, sloppy; 4 = pasty diarrhea; 5 = liquid diarrhea.

### Hematological parameters and immunological responses

It has been known that the frequency of diarrhea is correlated with the hematocrit values (25), and the serum hematological parameters are also associated with the stress and health status of the animals (26, 27). In addition, Bhattarai et al. (28) reported that red blood cells (RBC) and hemoglobin (HGB) were positively correlated with ADG in weaned pigs (28). However, there were no significant differences between dietary treatments of weaned piglets in all hematological parameters including RBC and HGB (Table 3). The blood parameter data for this study were within the normal range for the hematologic characteristics of weaned pigs as reported by previous studies (29).

**Table 3:** Effects of mixture of effective microorganisms on hematological parameters of weaning pigs.^a^

Item^b^	CON	MEM	SEM	*p*-value
WBC, 10^3^/μL				
Pre-weaning	19.01	22.13	1.76	0.226
Post-weaning	29.89	25.25	2.66	0.240
RBC, 10^6^/μL				
Pre-weaning	7.01	6.69	0.18	0.218
Post-weaning	7.12	7.20	0.22	0.965
HGB, g/dL				
Pre-weaning	11.24	10.47	0.38	0.174
Post-weaning	11.48	11.75	1.58	0.335
HCT, %				
Pre-weaning	41.83	40.84	1.22	0.572
Post-weaning	52.31	52.39	3.82	0.420
Lymphocyte, %				
Pre-weaning	48.51	45.58	2.92	0.487
Post-weaning	47.13	44.82	4.61	0.996
Monocyte, %				
Pre-weaning	3.41	4.34	0.58	0.268
Post-weaning	2.58	5.29	3.29	0.669
Eosinophil, %				
Pre-weaning	1.38	1.10	0.23	0.393
Post-weaning	0.32	0.55	2.33	0.347
Basophil, %				
Pre-weaning	0.43	0.53	0.08	0.374
Post-weaning	0.67	0.76	0.19	0.633

a Each value is the mean of 5 replicates.

b CON, a control diet without any treatments; MEM, a diet treated with mixture of effective microorganisms; SEM, standard error of mean; RBC, red blood cell; HGB, hemoglobin; HCT, packed cell volume (Hematocrit); PLT, platelets; MCV, mean corpuscular volume; MCH, mean corpuscular hemoglobin; MCHC, mean corpuscular hemoglobin concentration; Pre-weaning, the start of the experiment (D1); Post-weaning, the end of the experiment (D28).

In addition, no differences were observed between dietary treatments in the serum TNF-α, cortisol, and Immunoglobulin G, M, and A of weanling pigs during the experiment (Table 4). A number of previous studies have reported that cortisol concentrations, an indicator of weaning stress, increased over 7 days after weaning (30–32). In addition, pro-inflammatory cytokines such as TNF-α, which are closely related to weaning stress, are associated with transient inflammation in the intestine. Therefore, pro-inflammatory cytokines could cause negative effects for intestinal development and nutrient absorption in weaning piglets (33, 34). However, in this experiment, no differences were observed between dietary treatments in the serum TNF-α, and cortisol of weanling pigs during the experiment (Table 4). Similarly, no significant differences in immunoglobulin G, M, and A were found between dietary treatments in this experiment (Table 4), although, several other studies have reported that certain LABs, such as *Lactobacillus casei*, *Lactobacillus rhamnosus* and *Lactobacillus plantarum*, stimulated immune responses (35, 36). We speculated that these discrepancies between our results and others might be caused by the sampling time point.

**Table 4:** Effects of mixture of effective microorganisms on serum immune status of weanling pigs.^a^

Item^b^	CON	MEM	SEM	*p*-value
Cortisol, ng/mL				
Pre-weaning	5.12	3.14	0.52	0.053
Post-weaning	4.45	3.45	1.19	0.584
TNF-α, pg/mL				
Pre-weaning	78.51	89.70	4.97	0.187
Post-weaning	80.25	72.50	6.56	0.451
IgG, mg/mL				
Pre-weaning	639.22	401.18	76.73	0.093
Post-weaning	967.24	672.67	86.38	0.073
IgM, mg/mL				
Pre-weaning	1.58	3.58	0.48	0.069
Post-weaning	9.89	7.15	2.56	0.492
IgA, mg/mL				
Pre-weaning	7.77	9.14	2.36	0.703
Post-weaning	50.28	32.15	7.68	0.171

a Each value is the mean of 3 replicates.

b CON, a control diet without any treatments; MEM, a diet treated with mixture of effective microorganisms; SEM, standard error of mean; Pre-weaning, the start of the experiment (D1); Post-weaning, the end of the experiment (D28).

### Swine fecal microbial diversity

**Figure 1:**
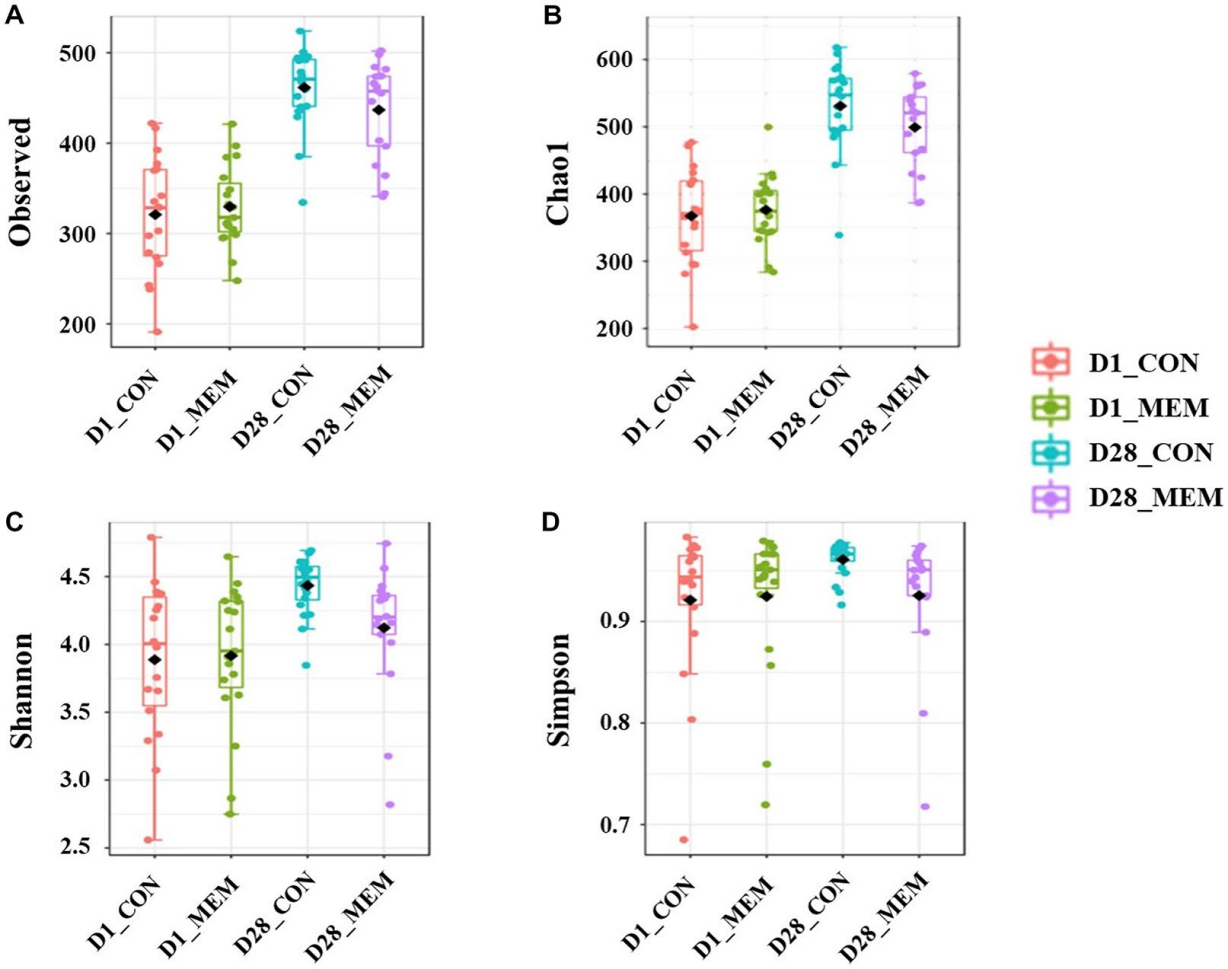
Box plots showing the alpha diversity indices of the pigs in CON and MEM on D1 and D28. **(A)** Number of observed OTUs, **(B)** Chao1 index, **(C)** Shannon index and **(D)** Simpson index. CON, a control diet without any treatments; MEM, a diet treated with mixture of effective microorganisms.

**Figure 2:**
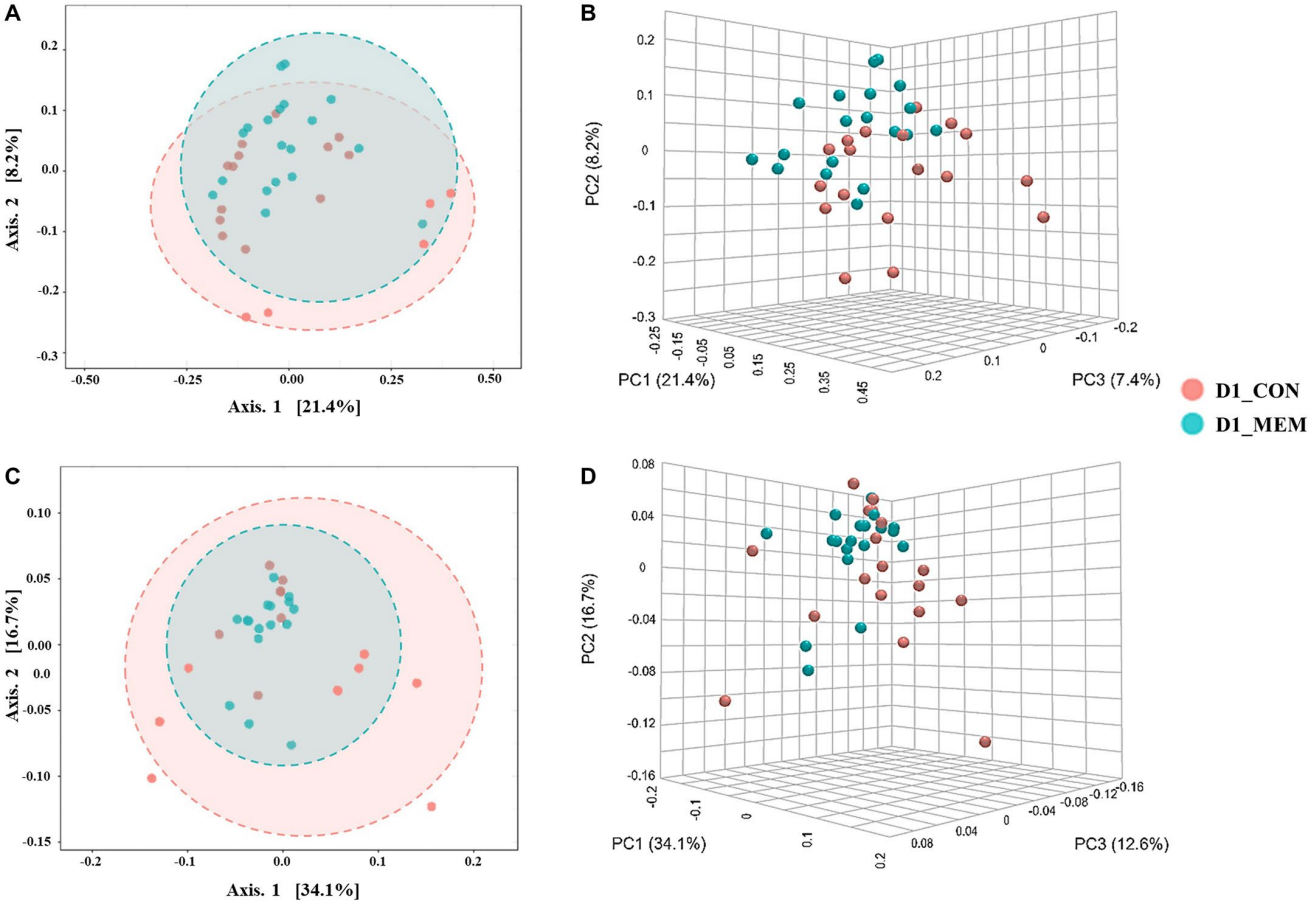
Beta diversity analysis of the pig gut microbiota of the pigs in CON and MEM on D1. Principal co-ordinates analysis (PCoA) plots based on the unweighted **(A, B)** and weighted **(C, D)** UniFrac distances of gut microbial communities (unweighted: R = 0.093567, *p* < 0.05; weighted: *R* = 0.060086, *p* < 0.05). Each symbol represents the microbiota from individual pig sample and was color coded according to dietary treatments (CON and MEM). The axes show the percent variation. CON, a control diet without any treatments; MEM, a diet treated with mixture of effective microorganisms.

**Figure 3:**
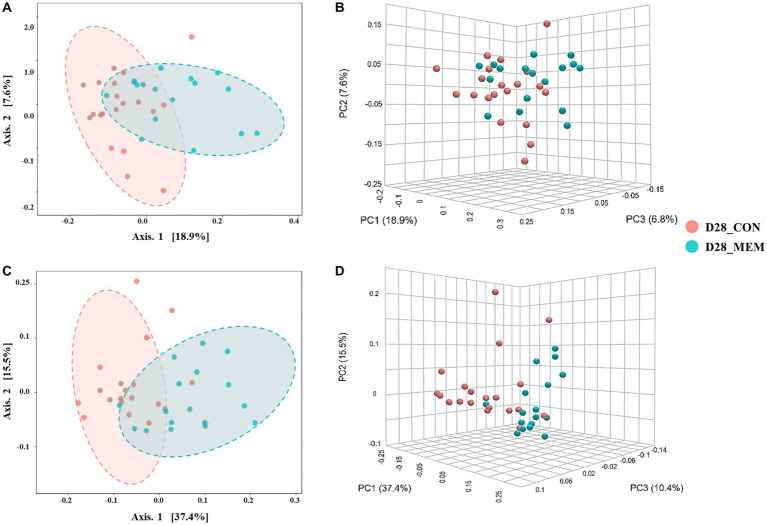
Beta diversity analysis of the pig gut microbiota of the pigs in CON and MEM on D28. Principal co-ordinates analysis (PCoA) plots based on the unweighted **(A, B)** and weighted **(C, D)** UniFrac distances of gut microbial communities (unweighted: *R* = 0.243, *p* < 0.001; weighted: *R* = 0.29356, *p* < 0.001). Each symbol represents the microbiota from individual pig sample and was color coded according to dietary treatments (CON and MEM). The axes show the percent variation. CON, a control diet without any treatments; MEM, a diet treated with mixture of effective microorganisms.

We performed 16S rRNA gene analysis on the fecal samples collected on the first and final day of the experiment (post weaning D1 and D28). The microbial diversity indices are presented in Figure 1. No differences in Chao1, Shannon, and Simpson diversity indices were observed between the MEM and CON. The visualization of the relative distances of microbial communities between the CON and MEM using a principal coordinate analysis (PCoA) plot are shown in Figures 2, 3.

At the first day of the experiment, the PCoA plots based on the unweighted and weighted UniFrac distances revealed no differences of microbial communities between dietary treatments (Unweighted: *R* = 0.09567. *p* < 0.05; Weighted: *R* = 0.060086, *p* < 0.05) (Figure 2). However, microbial populations were clustered into two distinct groups at the end of the experiment (Unweighted: *R* = 0.243. *p* < 0.001; Weighted: *R* = 0.29356, *p* < 0.001) (Figure 3). Because the pigs were housed under the same conditions and fed the same feed, it is reasonable to speculate that the differences of microbial communities between dietary treatments could be the effects of the MEM.

### Taxonomic classification of the sequences

**Figure 4:**
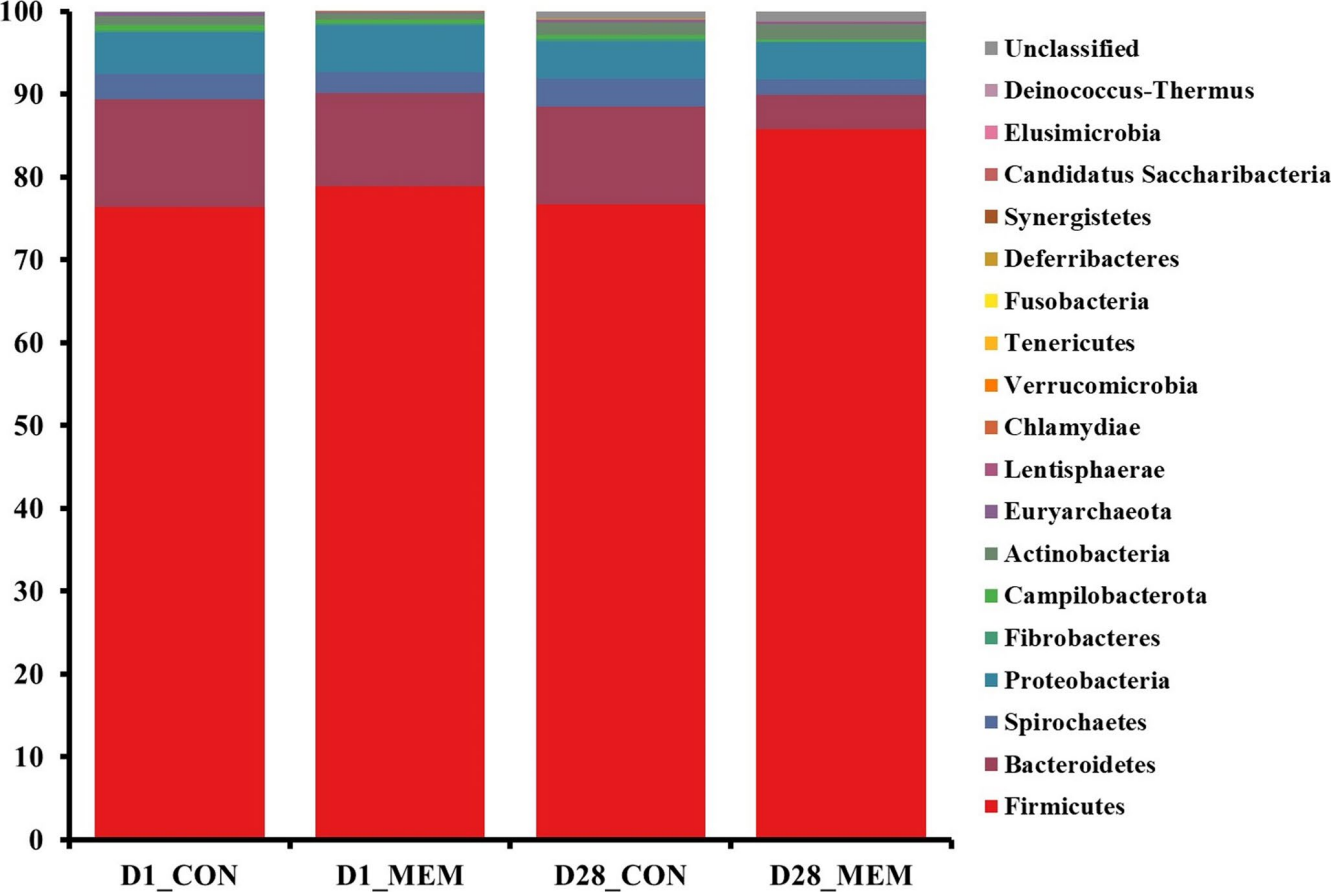
Stacked bar plots of the relative abundance of gut microbial communities at the phylum levels in weanling pigs on D1 and D28. CON, a control diet without any treatments; MEM, a diet treated with mixture of effective microorganisms.

**Figure 5:**
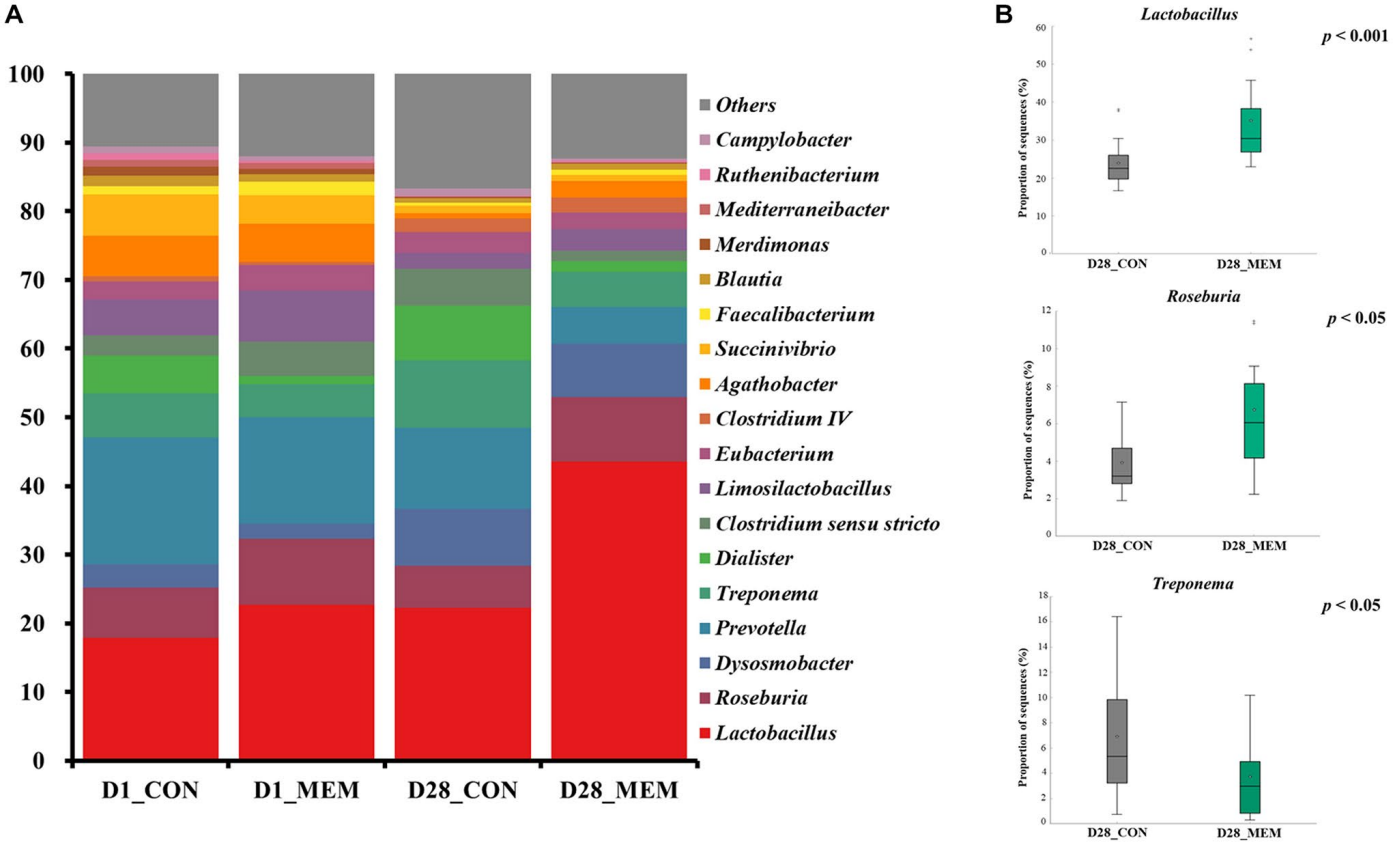
Stacked bar plots of the relative abundance of gut microbial communities at the genus levels in weanling pigs on D1 and D28. Relative abundances of the sequences at the genus level **(A)**, Relative abundances of *Treponema, Lactobacillus,* and *Roseburia* on d28 **(B)**. CON, a control diet without any treatments; MEM, a diet treated with mixture of effective microorganisms.

The relative abundances of the bacterial taxa at the phylum and genus levels for all sequences are shown in Figures 4, 5, respectively. There were no differences in the relative fecal microbial compositions between dietary treatments at the phylum level during the experiment (Figure 4). The three phyla Firmicutes, Bacteriodetes and Proteobacteria were sequentially dominant in both dietary treatments, of which Firmicutes, Bacteriodetes accounted for approximately 90% of the total sequence reads. This result is consistent with previous studies on the swine gut microbiota, the two most abundant taxa at the phylum level were Firmicutes and Bacteroidetes (37, 38). The result of this experiment showed that the ratio of Firmicutes to Bacteroidetes (FB ratio) of the two dietary treatments on the first day of the weaning was similar, however, the FB ratio of the MEM at 4 weeks after treatment was 2 times higher than that of the CON. This is consistent with a previous study that reported an association between improved weight gain and the higher FB ratio (39). They reported that the higher the FB ratio produced the more short chain fatty acids during microbial metabolism, which exhibit anti-inflammatory and antioxidant properties in the porcine intestinal tract, which is associated with weight gain (40, 41). The difference in fecal microbial compositions between CON and MEM was confirmed at the genus level (Figure 5). The relative abundances of *Treponema, Lactobacillus,* and *Roseburia* were not significantly differ between dietary treatments on D1. Regarding the relative abundance of *Treponema* that is known as a potential pathogen and is mainly responsible for intestinal inflammation, the MEM had significantly lower (*p* < 0.05) *Treponema* than CON on D28 (Figure 5B). Whereas MEM had significantly higher (*p* < 0.001) *Lactobacillus* which helps prevent infection and improve growth performance (42), compared to the CON on D28 (Figure 5B). Also, it was confirmed that the relative abundance of *Roseburia*, which produces short chain fatty acids and acts as an anti-inflammatory agent was significantly higher in the MEM than the CON (*p* < 0.05) on D28 (Figure 5B) (43–46).

The results of alpha diversity analyses showed no significant changes in the gut microbial richness between dietary treatments. However, the PCoA plot presented differences in the gut microbial composition and their relative abundances between CON and MEM on D28. As such, relative abundances of *Treponema, Lactobacillus,* and *Roseburia* shifted over time. Overall, these results indicate that the MEM shifted the gut microbial communities in the studied pigs.

## Conclusion

Overall, our data showed that *L. casei* and *S. cerevisiae* mixture improved the growth rate of weaned pigs by shifting their gut microbiome. Mixture of *L. casei* and *S. cerevisiae* increased the abundances of beneficial microbes including *Lactobacillus* and *Roseburia*. The findings of this study suggested that the mixed effective microorganisms could promote growth performance through modulation of gut microbiota in pigs.

## Data availability statement

The datasets presented in this study can be found in online repositories. The names of the repository/repositories and accession number(s) can be found below: https://www.ncbi.nlm.nih.gov/, PRJNA905856.

## Ethics statement

The animal study was reviewed and approved by Institutional Animal Care and Use Committee of the Dankook University, Cheonan, South Korea (approval no. DKU-21-040). Written informed consent was obtained from the owners for the participation of their animals in this study.

## Author contributions

SaK, MS, JC, and HK: conceptualization. MS: data curation. HD, JK, SP, SR, YI, and ShK: formal analysis. EK, ShK, and YI: methodology. HD: resources. GK: software. JC: supervision. SaK, YI, and MS: validation. JHC: visualization. ShK, JK, JC, and MS: writing – original draft. HK: writing – review and editing. All authors contributed to the article and approved the submitted version.

## Funding

This work was carried out with the support of ‘Basic Science Research Program funded by the Ministry of Education (2021R1I1A3059910)’ and ‘Cooperative Research Program for Agriculture Science & Technology Development (PJ0162232022)’ Rural Development Administration, Korea.

## Conflict of interest

SaK was employed by BRD Korea Corp.

The remaining authors declare that the research was conducted in the absence of any commercial or financial relationships that could be construed as a potential conflict of interest.

## Publisher’s note

All claims expressed in this article are solely those of the authors and do not necessarily represent those of their affiliated organizations, or those of the publisher, the editors and the reviewers. Any product that may be evaluated in this article, or claim that may be made by its manufacturer, is not guaranteed or endorsed by the publisher.

## Abbreviations

ADFI: Average daily feed intake
ADG: Average daily gain
BW: Body weight
EDTA: Ethylenediaminetetraacetic acid
FB ratio: ratio of Firmicutes to Bacteroidetes
GIT: Gastrointestinal tract
HCT: Hematocrit
HGB: Hemoglobin
MCH: Mean corpuscular hemoglobin
MCHC: Mean corpuscular hemoglobin concentration
MCV: Mean corpuscular volume
PLT: Platelets
SEM: Standard error of mean
TNF-α: Tumor necrosis factor-α
RBC: Red blood cell
